# Osmolarity influences chondrocyte repair after injury in human articular cartilage

**DOI:** 10.1186/s13018-015-0158-z

**Published:** 2015-01-28

**Authors:** Yuelong Huang, Yujun Zhang, Xiaoquan Ding, Songyang Liu, Tiezheng Sun

**Affiliations:** Arthritis Clinic and Research Center, Peking University People’s Hospital, No. 11 Xizhimen South Street, Xicheng District Beijing, 100044 China; Clinic Molecular Institute, Peking University People’s Hospital, No. 11 Xizhimen South Street, Xicheng District Beijing, 100044 China

**Keywords:** Osmolarity, Irrigation solution, Chondrocyte, Articular cartilage

## Abstract

**Background:**

The purpose was to determine the influence of irrigation solution osmolarity on articular chondrocytes survival and metabolic state following mechanical injury.

**Methods:**

Osteochondral explants were harvested from patients undergoing total knee arthroplasty for osteoarthritis and then cut through their full thickness to establish mechanical injury models. Cartilage explants were incubated in irrigation solutions (saline and balanced salt) with different osmolarities (180, 280, 380, 580 mOsm/L) for 2 h. The percentage of cell death (100 × number of dead cells/number of dead and live cells) was quantified with the laser confocal microscopy. The terminal deoxynucleotidyl transferase-mediated dUTP nick-end labeling (TUNEL) assay was performed to detect apoptosis index of injured cartilage. The contents of proteoglycan elution were determined by spectrophotometer at 530 nm, and HIF-1α and type II collagen mRNA yields were quantified with real-time PCR.

**Results:**

*In situ* dead chondrocytes were mainly localized to the superficial tangential region of injured cartilage edge after mechanical injury. The percentage of cell death was decreased, and proteoglycan elution was gradually reduced with the increasing of osmolarity. The apoptosis indices of TUNEL assay in different osmolarities had no significant difference (*P* = 0.158). HIF-1α and type II collagen mRNA yields were the least for chondrocytes exposed to 180 mOsm/L medium and were the greatest for chondrocytes exposed to 380 mOsm/L medium. Compared with the saline group, the cell death of superficial zone was significantly decreased (*P* = 0.001) and contents of proteoglycan elution were also significantly decreased (*P* = 0.045) in the balanced salt. HIF-1α (*P* = 0.017) and type II collagen (*P* = 0.034) mRNA yields in the chondrocytes exposed to the balanced salt were significantly more than the saline group.

**Conclusion:**

The osmolarity of irrigation solutions plays an important role in the survival and metabolic state of chondrocytes following mechanical injury, and the chondrocyte death is not caused by apoptosis. Increasing osmolarity of irrigation solutions may be chondroprotective with decreasing the chondrocyte death, reducing inhibition of metabolism and proteoglycan elution, ultimately preventing cartilage degeneration and promoting integrative repair.

## Background

Numerous arthroscopic procedures involve wounding articular cartilage, for instance, during osteochondral harvest for transplantation or debridement of chondral defect [1]. Such surgical injury results in a region of chondrocyte death and extracellular matrix degradation at the wounded edge that limits successful integrative cartilage repair. During articular surgical procedure, synovial fluid is replaced by the irrigation solutions for visualization of the joint, with the osmolarity ranging from 250 to 300 mOsm/L. However, the osmolarity of normal human synovial fluid is approximately 400 mOsm/L [2]. *In situ* chondrocytes therefore experience a marked decrease in extracellular osmolarity during the arthroscopic surgery. Nevertheless, *in situ* chondrocytes are osmotically sensitive and react to change in extracellular osmolarity [3,4]. In an animal model, the osmolarity of the extracellular matrix has a significant impact on chondrocytes following mechanical injury that a decrease in extracellular osmolarity increases chondrocyte death, whereas an increase in extracellular osmolarity has a protective effect [5]. However, only one report from Amin et al. talked about the influence of osmolarity changes on human articular cartilage [6], and the mechanism responsible for influence of the medium osmolarity on the survival of chondrocyte following mechanical injury remains unclear. Modulating the osmolarity to find appropriate properties of irrigation solutions will minimize chondrocyte death from the mechanical injury and be beneficial to retain a viable cell population within the wounded edge, which is favorable of better cartilage repair.

Mechanical injury model was established with human articular cartilage and was incubated in irrigation solutions of different osmolarities for about 2 h, and then, we determined the influence of irrigation solution osmolarity on articular chondrocyte survival, proteoglycan elution, and metabolic state following mechanical injury.

## Materials and methods

### Cartilage preparation

Human osteochondral materials were obtained from 20 patients undergoing total knee arthroplasty (TKA) for osteoarthritis. The grade 0 or 1 cartilage was selected under macroscopic and microscopic observation, according to International Cartilage Repair Society (ICRS) cartilage tissue scores [4,7]. All osteochondral tissues were immediately placed in serum-free Dulbecco’s Modified Eagle’s Medium (DMEM; Invitrogen Ltd, USA) and were used within 1 h after resection. The mean age of the patients with osteoarthritis was 69 years old (range: 65–76 years), and there were 3 males and 17 females. Written informed consent for the study was obtained from all of the patients. The research protocol was approved by the Institutional Review Board of the Peking University People’s Hospital.

### Mechanical injury model

**Figure 1:**
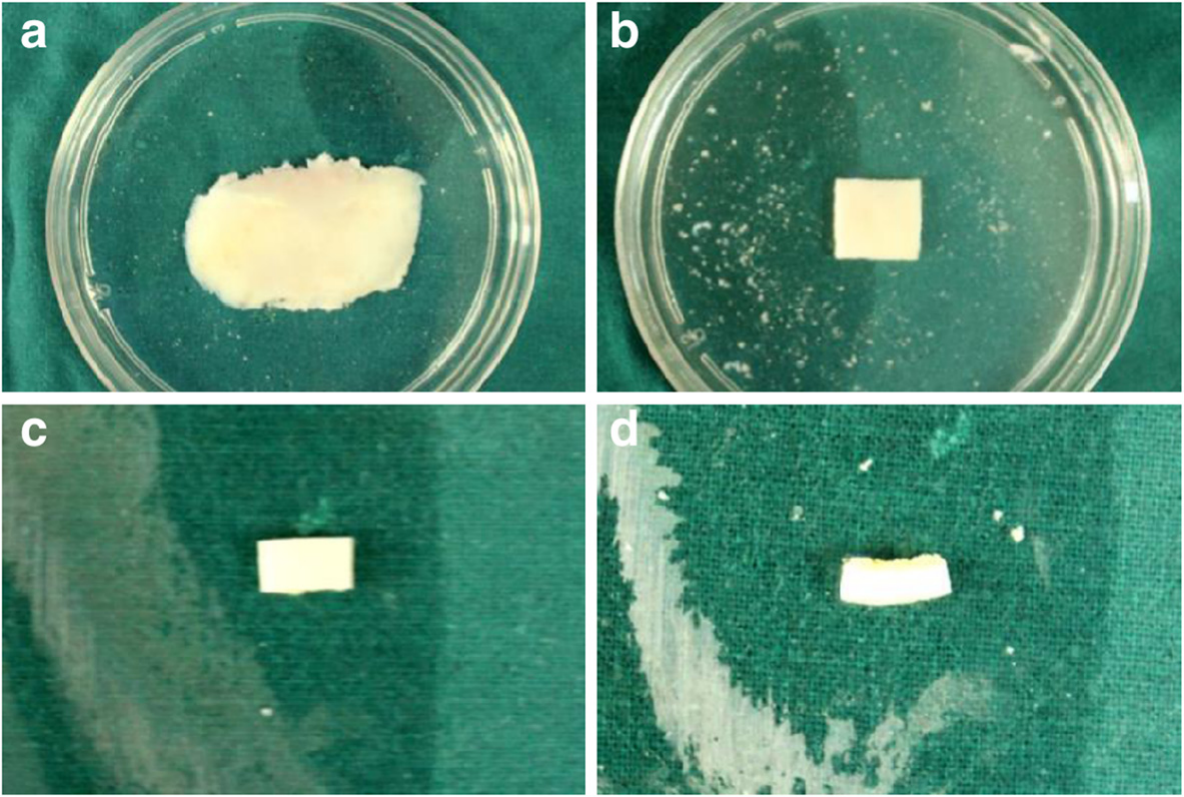
**Making the human articular cartilage mechanical injury model. (a)** Distal articular surface of femoral lateral condylar of the knee joint. **(b)** Osteochondral tissue consisting of the full-thickness cartilage with subchondral bone. **(c)** The osteochondral tissue is vertically cut into explants to produce mechanical injury model at the margin. **(d)** The lateral view of mechanical injury model on osteochondral explant.

Human osteochondral tissues from lateral femoral condyle of eight patients were vertically cut through their full thickness with a fresh no. 24 scalpel blade to produce mechanical injury models at the edge of the scalpel cut. The osteochondral tissues were cut into 5 × 3 mm rectangular osteochondral explants, consisting of the full-thickness cartilage with subchondral bone (approximately 2 mm) (Figure 1). Osteochondral explants were randomly incubated in irrigation solutions with different osmolarities (180, 280, 380, and 580 mOsm/L) and primary solutions (saline and balanced salt) at 5% CO_2_, 37°C for 1.5 h. Saline was obtained from China Resources Pharmaceutical Company (National Drug Approval No: H20023300, major ingredients: sodium chloride, 154 mmol/L); balanced salt was obtained form Tiancheng Pharmaceutical company (National Drug Approval No: H20130032; major ingredients: sodium chloride, 89 mmol/L; sodium gluconate, 23 mmol/L; potassium chloride,5 mmol/L; and magnesium chloride, 1.48 mmol/L). The osmolarity of the solutions was adjusted to 180, 280, 380, and 580 mOsm/L by adding distilled water or sucrose respectively and measured with a freezing point osmometer (Advanced Micro Osmometer, Model 3300; Vitech Scientific Ltd, UK).

After that, explants were exposed to 10 μM fluorescent probes 5-chloromethyl-fluorescein diacetate (CMFDA; Invitrogen, USA) and 10 μΜ propidium iodide (PI; Invitrogen, USA) for additional 30 min of incubation to label live and dead cells, respectively. CMFDA can freely pass through the cell membrane and produce impermeable 5-chloromethyl-furfural (5-CMF) after an enzymatic reaction, and the green fluorescence is stimulated so that the cytoplasm is colored green that represents the living cells. PI has a positive charge and can only penetrate dead cell membrane and combine with a nucleus that is colored red [8]. Finally, the explants were transferred to 10% formalin for fixation and stored in 4°C phosphate-buffered saline before microscopy.

Using a confocal laser scanning microscope (Leica TCS SP8, Germany), optical sections were acquired in the coronal plane from mechanical injury models (superficial and deep), counting the number of chondrocyte death based on fluorescence and then quantifying the percentage of cell death. The microscope was fitted with (×10) objective and obtained the fluorescence channels emitted from CMFDA (λex = 488 nm and λem = 517 nm) and PI (λex = 543 nm and λem = 650 nm), respectively. By moving the focal plane into the depth of the cut surface, consecutive series of optical sections were acquired at intervals of 10 μm in the coronal planes, to a depth of approximately 150 μm, and finally made the reconstructed three-dimensional image. By moving the focal point to regions of interest, the three-dimensional image was obtained from different coronal planes and the total volume of each region of interest (ROI) for subsequent quantitative analysis was 1,500 × 600 × 150 μm^3^ (*x*-*y*-*z* axes, respectively). Using Leica QWin Image analysis software (Leica Corporation, Germany), the number of living cells and dead cells was quantified, and then the percentage of cell death was calculated.

### TUNEL assay

The cartilage explants from five patients were obtained using the same procedure as described above and randomly exposed to different osmolarity solutions for 2 h. Terminal deoxynucleotidyl transferase-mediated dUTP nick-end labeling (TUNEL) assay was used to detect apoptotic cells, with a TUNEL Assay Kit (Roche, Germany). Cells with sepia nucleus were assessed as positive. The percentage of apoptotic cells (apoptosis index) was evaluated by randomly selected five fields (×100) in each section from different osmolarity groups.

### Proteoglycan elution from cartilage

The osteochondral explants from lateral femoral condyle of seven patients were obtained using the same procedure as described above and then were randomly incubated at 5% CO_2_, 37°C for a further 2 h culture in the respective solutions with different osmolarities. An aliquot of the supernatant extracted from the medium was used to determine the contents of proteoglycan elution by measuring the absorbance at 530 nm on a spectrophotometer using the dimethyl-methylene blue method [9]. The concentration of proteoglycan was measured by the use of chondroitin sulfate to draw a calibration curve.

### Primary chondrocytes culture

Articular cartilage from lateral tibial plateau of fifteen patients was cut into about 1 mm^3^ fragments and digested with 0.2% protease (Sigma, USA) for 1 h and then were further digested with 0.15% collagenase II (Invitrogen, USA) in DMEM for overnight. Chondrocytes were collected and resuspended in 15% FBS-DMEM medium containing 1 g/L penicillin-streptomycin (Invitrogen, USA). After confluence, the cultured cells were detached using 2.5 g/L trypsin (Invitrogen, USA), seeding at the density of 3 × 10^4^/cm^2^.

### Real-time PCR

5 × 10^6^ chondrocytes of the second passage were incubated in respective solutions with different osmolarities for further 2 h. Total RNA was extracted from chondrocytes with Total RNA kit (Omega, USA). The first strand complementary DNA (cDNA) was synthesized from 1 μg isolated mRNA yields using reverse transcription kit (TOYOBO: FSQ-201, JAPAN) and used as templates for real-time PCR. The expression of mRNA yields was determined quantitatively using SYBR Green mix (TOYOBO: QPS-201, JAPAN) with Bio-Rad qPCR (Bio-Rad Laboratory, USA) instrument. β-actin was housekeeping gene, and primer’s sequences of the targeted genes were listed (Table 1).

**Table 1:** **Primer’s sequences of the targeted genes used for real-time PCR**

**Target gene**	**Orientation**	**Primer sequence (5′-3′)**
β-actin	Forward	CACCATTGGCAATGAGCGGTTC
	Reverse	AGGTCTTTG CGGATGTCCACGT
HIF-1α	Forward	TATGAGCCAGAAGAACTTTTAGGC
	Reverse	CACCTCTTTTGGCAAGCATCCTG
Collagen II	Forward	CCTGGCAAAGATGGTGAGACAG
	Reverse	CCTGGTTTTCCACCTTCACCTG

The samples underwent 40 cycles consisting of the following steps: denaturation at 94°C for 20 s, annealing at 65°C for 20 s, and extension at 72°C for 30 s. Data was analyzed by the use of ΔΔCt, and results were expressed as 2-ΔΔCt.

### Statistical analysis

All statistical analyses were performed using SPSS version 19.0, and data was expressed as $\overline{x}\pm s.$ Analyses of variance (ANOVA) were conducted to compare trends among irrigation solutions with different osmolarities, and Student’s *t* test was conducted to compare trends between saline and balanced salt groups, with the level of significance set at *p* < 0.05.

## Results

### Cell death in cartilage mechanical injury model

**Figure 2:**
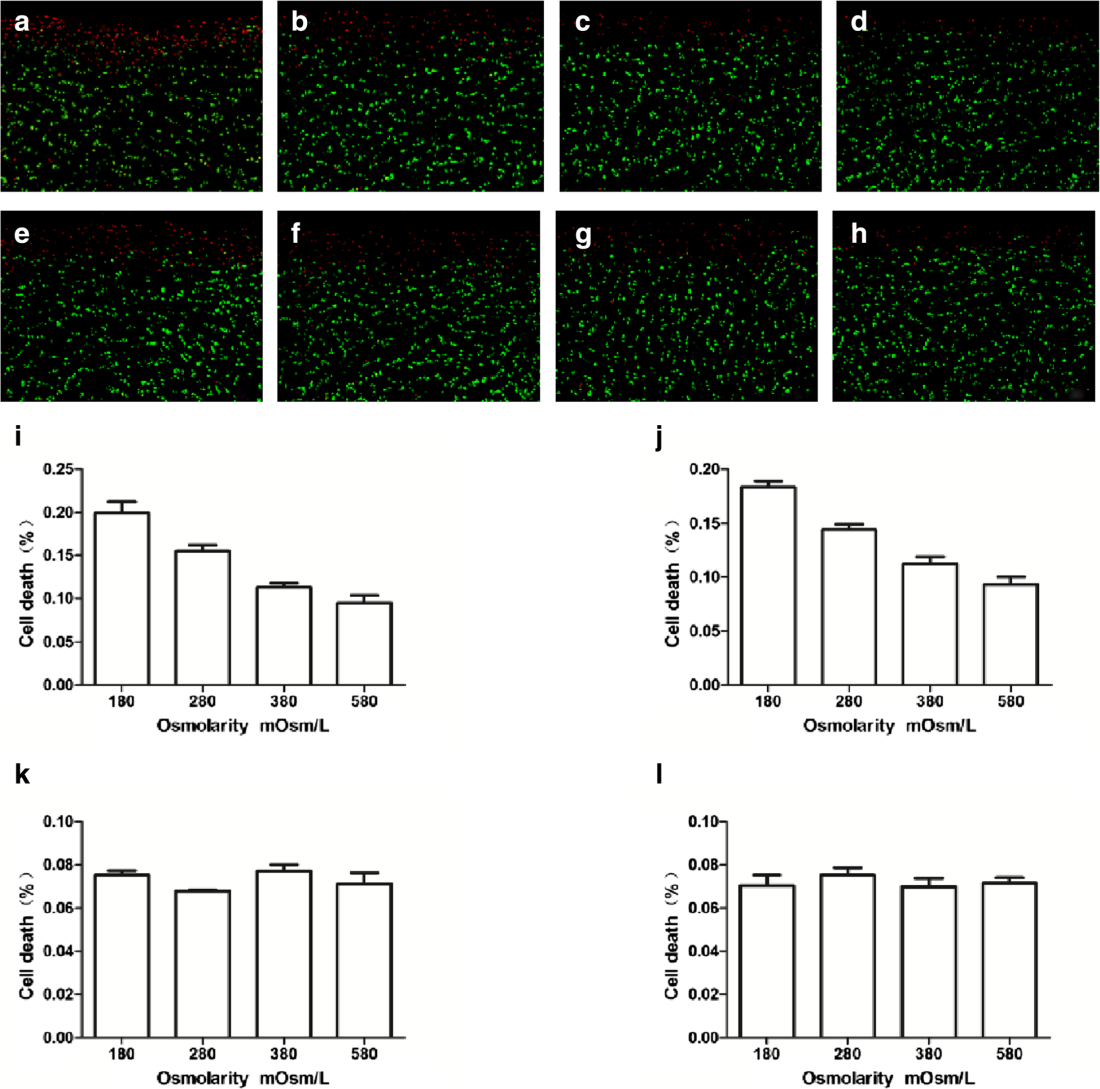
**Effect of different osmolarity irrigation solutions on cell viability and cell death.** Using laser confocal microscopy to observe the survival of the chondrocytes, red stands for dead cells, green stands for living cells **(a–d)** saline; **(e–h)** balanced salt; **(a, e)** 180 mOsm/L; **(b, f)** 280 mOsm/L; **(c, g)** 380 mOsm/L; and **(d, h)** 580 mOsm/L. Effects of different osmolarity irrigation solutions on cell death in the superficial or deep zone. **(i, k)** Saline; **(j, l)** balanced salt; **(i, j)** superficial zone; and **(k, l)** deep zone (*n* = 6 for each osmolarity; ANOVA tests for superficial zone: saline: *P* = 0.001; balanced salt: *P* = 0.001; deep zone: saline: *P* = 0.93; balanced salt: *P* = 0.92).

**Figure 3:**
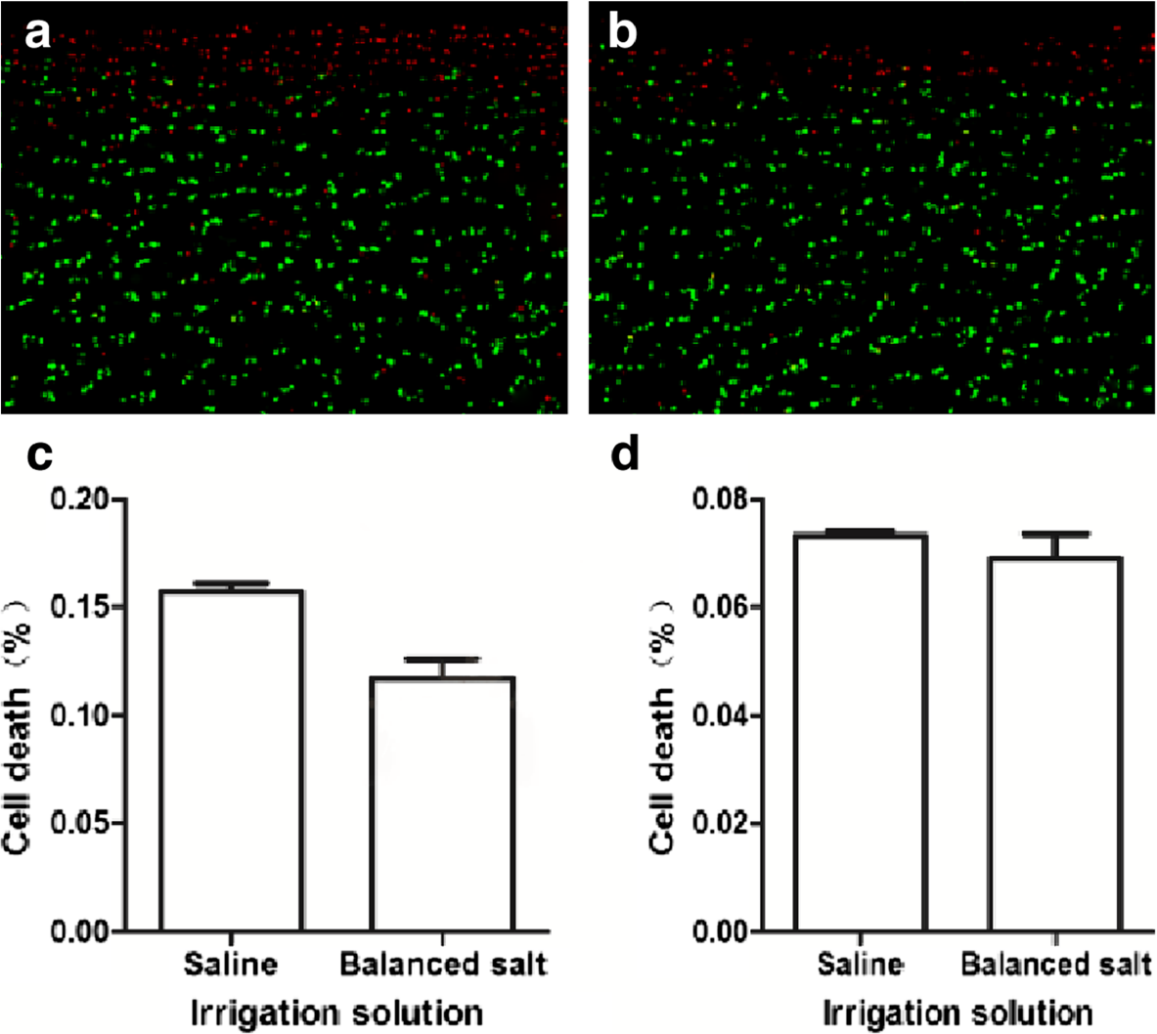
**Effect of different primary solutions on cell death in superficial and deep zone.** In the human articular cartilage mechanical injury model **(a)** saline (superficial zone); **(b)** balanced salt (superficial zone); the percentage of cell death in balanced salt and saline solutions **(c)** superficial zone; and **(d)** deep zone (*n* = 6 for each solution; *t* tests for superficial zone: *P* = 0.001; deep zone: *P* = 0.24).

The mechanical injury model demonstrated that *in situ* dead chondrocytes were mainly localized to the superficial zone orientated tangentially to the articular surface (Figure 2a–h) with the change of medium osmolarity. The percentage of cell death was gradually decreased with the increasing of osmolarity in the superficial zone (saline: *P* = 0.001; balanced salt: *P* = 0.001) (Figure 2i, j). There was no statistically significant difference of cell death in deep zone of articular cartilage (saline: *P* = 0.93; balanced salt: *P* = 0.92) (Figure 2k, l). Compared with the saline of primary solutions, the dead cells in the superficial zone were statistically significantly lower (*P* = 0.001) in the balanced salt (Figure 3a–c); however, there was no statistically significant difference (*P* = 0.24) in the deep zone cells (Figure 3d).

### Chondrocyte apoptosis in cartilage

**Figure 4:**
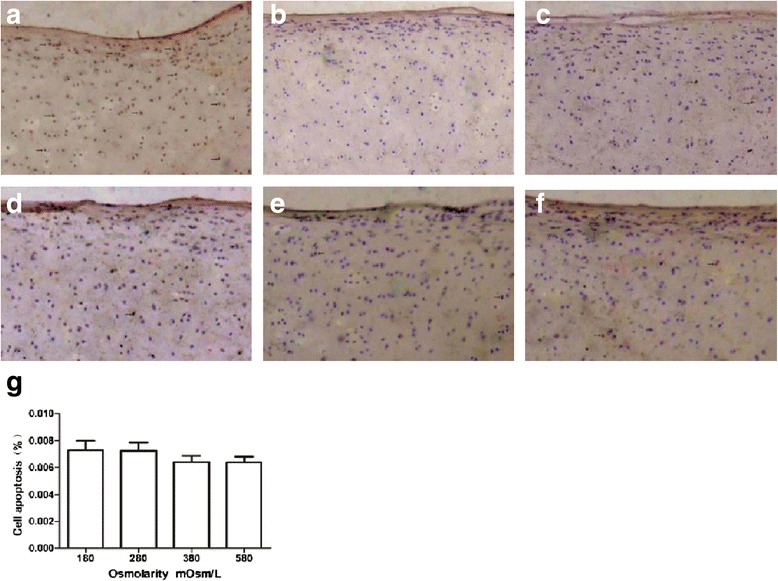
**Effect of different osmolarities of saline on chondrocytes apoptosis and representative sections from TUNEL assay.** In the human articular cartilage mechanical injury model, sepia stands for apoptotic cells **(a)** positive control; **(b)** negative control; **(c)** 180 mOsm/L; **(d)** 280 mOsm/L; **(e)** 380 mOsm/L; **(f)** 580 mOsm/L; and **(g)** the percentage of apoptotic cells (*n* = 6 for each osmolarity, ANOVA test, *P* = 0.158).

The chondrocyte apoptosis detected by TUNEL assay was shown that fewer positive cells were observed in different osmolarity groups compared with the positive control (Figure 4a–f). Less than 1% of chondrocytes were positive in apoptotic assay of different osmolarity groups after 2 h. The apoptosis indices in different osmolarities (180, 280, 380, and 580 mOsm/L) were 0.73%, 0.72%, 0.65%, and 0.64%, respectively, and there was no statistically significant difference among them (*P* = 0.158) (Figure 4g).

### Proteoglycan elution

**Figure 5:**
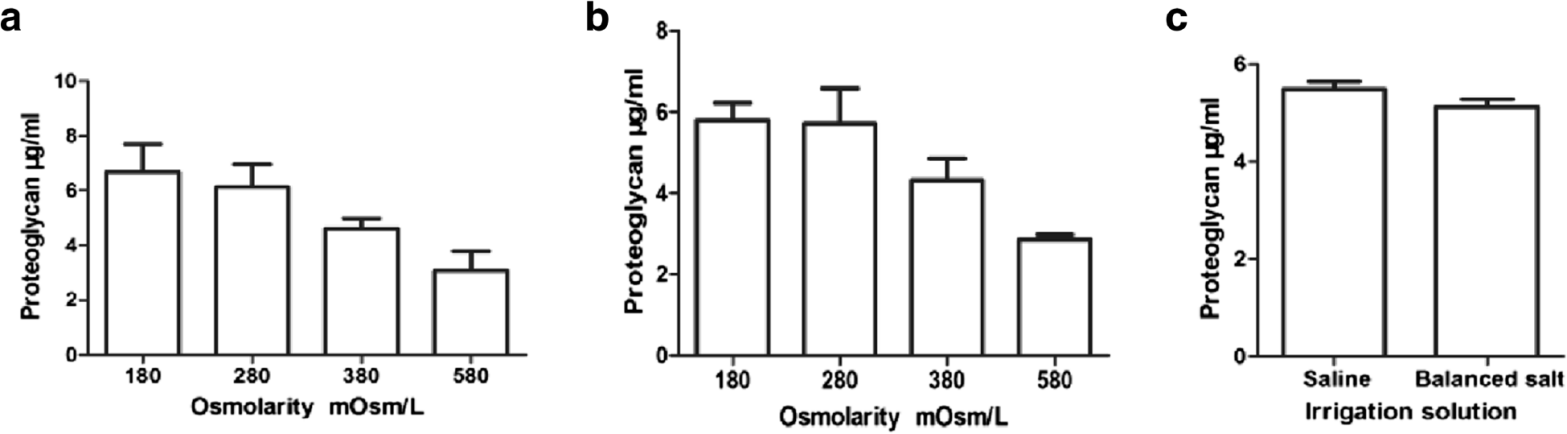
**Effect of different osmolarity irrigation solutions on proteoglycan elution.** In the human articular cartilage mechanical injury model **(a)** saline in different osmolarities; **(b)** balanced salt in different osmolarities; and **(c)** comparison of proteoglycan elution between the saline and balanced salt. (*n* = 6 for each osmolarity and solution; ANOVA tests for saline: *P* = 0.002; balanced salt: *P* = 0.001; *t* test of saline vs balanced salt: *P* = 0.045).

Considerable amounts of proteoglycan were washed out from the cartilage matrix for explants exposed to the different osmolarity medium. It was noted that irrigation solutions of osmolarity 180 mOsm/L extracted far more proteoglycan from the cartilage matrix than other osmolarity medium, no matter in the saline or the balance salt solutions. With increasing of the osmolarity, the total contents of proteoglycan elution were gradually decreased (saline: *P* = 0.002; balanced salt: *P* = 0.001) (Figure 5a, b), where there was no statistically significant difference between 180 and 280 mOsm/L (saline: *P* = 0.39; balanced salt: *P* = 0.64). Compared with the saline of primary solutions, proteoglycan elution was statistically significantly decreased (*P* = 0.045) in balanced salt solutions (Figure 5c).

### Expression of HIF-1α and type II collagen mRNA in chondrocytes

**Figure 6:**
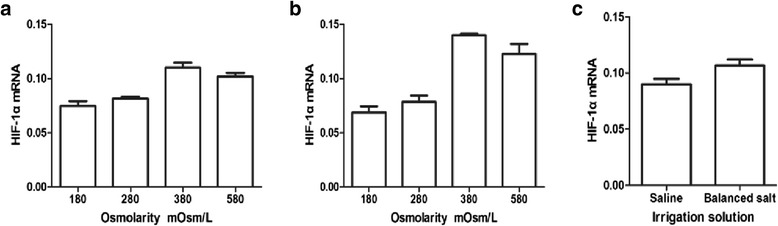
**Effect of different osmolarity irrigation solutions on expression of HIF-1α** **mRNA.** In the human articular chondrocytes with real-time PCR **(a)** saline in different osmolarities; **(b)** balanced salt in different osmolarities; and **(c)** comparison of HIF-1α mRNA between saline and balanced salt (ANOVA tests for saline: *P* = 0.001; balanced salt: *P* = 0.001; *t* test for saline vs balanced salt: *P* = 0.017, *n* = 3 for each osmolarity and solution; repeat for three times, the figure shows only one representative).

**Figure 7:**
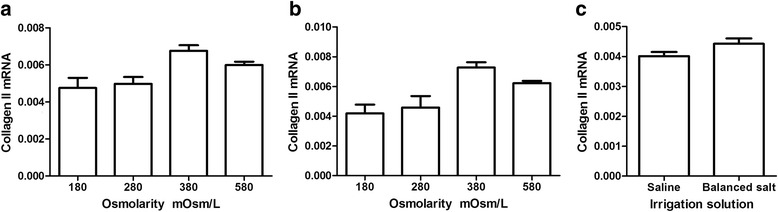
**Effect of different osmolarity irrigation solutions on expression of type II collagen mRNA.** In the human articular chondrocytes with real-time PCR **(a)** saline in different osmolarities; **(b)** balanced salt in different osmolarities; and **(c)** comparison of type II collagen mRNA between saline and balanced salt (ANOVA tests for saline: *P* = 0.001; balanced salt: *P* = 0.001; *t* test for saline vs balanced salt: *P* = 0.034, *n* = 3 for each osmolarity and solution, repeat for three times, the figure shows only one representative).

After incubation of chondrocytes in different solutions (saline, balanced salt), along with increasing of the osmolarity, HIF-1α and type II collagen mRNA yields were the least for chondrocytes exposed to 180 mOsm/L medium and were the greatest for chondrocytes exposed to 380 mOsm/L medium and then decreased with increasing of osmolarity (saline: *P* = 0.001; balanced salt: *P* = 0.001) (Figure 6a, b; Figure 7a, b). However, there was no statistically significant difference between the 180 and 280 mOsm/L groups (saline: *P* = 0.058; balanced salt: *P* = 0.087). HIF-1α (*P* = 0.017) and type II collagen mRNA (*P* = 0.034) yields from the chondrocytes of the balanced salt solutions were statistically significantly more than the saline (Figures 6c and 7c).

## Discussion

Sharp articular cartilage injury with a scalpel is a reproducible model *in vitro*, which allows spatial quantification of *in situ* chondrocyte death within all zones of the injured cartilage to predict the responses of sharply wounded articular cartilage during articular surgery [3,10]. Scalpel-cut explants are as models to study the effect of medium osmolarity on chondrocyte death for the reason of effectively avoiding the variation in matrix damage and chondrocyte death [11]. The duration of explant exposed to a medium with altered osmolarity (about 2 h) was simulated to the time during arthroscopic surgery. Amin et al. proved that there was no increase in chondrocyte death from 2.5 h to 7 days in animal model [12], suggesting that early use of irrigation solutions (about 2 h) played an important role in chondrocyte survival. The integrity of superficial zone and survival of *in situ* chondrocytes are significantly important for maintaining the biomechanical properties of cartilage and preventing cartilage degeneration [1]. Our findings show that chondrocytes in the superficial zone are the most susceptible to the full-thickness cartilage injury and dead cells of higher density are mainly localized to the superficial zone of the scalpel-cut edge. The results show similar trends with Amin et al.’s reports in human articular cartilage [6]; moreover, we further tested effects of osmolarity on chondrocyte survival after mechanical injury in different irrigation solutions with osmolarity gradients, elucidated the mechanism with detecting apoptosis index, exploring the influences of osmolarity on cartilage matrix proteoglycan elution and *in vitro* chondrocyte metabolism.

The accurate mechanism responsible for the impact of the medium osmolarity on the chondrocyte survival following mechanical injury remains unclear. Bush et al. pointed out that the volume change of chondrocytes exposed to the mechanical stress had a significant effect on cell survival [13]. Chondrocyte morphology and metabolism was determined in the cultured osmolarity medium of 280 ~ 580 mOsm/L *in vitro*, Xu et al. found that the cell volume swelled 20% in 280 mOsm/L and shrinked 15% in 580 mOsm/L [10]. Hyposmolarity makes cell swelling and hyperosmolarity makes cell shrinkage, by which swelling chondrocytes of osmotic responses become more susceptible to mechanical injury [14]. Those cell death induced by mechanical injury may be the result of necrosis, apoptosis, or other forms. Our data demonstrated that less than 1% of these chondrocytes were positive in apoptosis assay in different osmolarity groups, which indicated that the cell death in the cartilage following mechanical injury was mainly not caused by apoptosis.

The elastic component is decreased due to the proteoglycan elution, affecting the structure of cartilage, where the viability of chondrocytes is more susceptible to mechanical injury. Proteoglycan is an important component in the cartilage matrix accounting for about 7%–10% of the cartilage that plays an important role in stress load dispersion in articular cartilage. The matrix proteoglycan can easily diffuse through the intact cartilage surface and appear in the incubation medium under certain conditions [14,15]. The extracellular osmolarity is greater with precise values determined by the proteoglycan concentration when proteoglycan is released from the collagen meshwork to the medium [16,17]. Our study finds that amounts of proteoglycan are washed out from the cartilage matrix and proteoglycan elution decreases with the increasing medium osmolarity. Reduction of proteoglycan in cartilage matrix can affect the interaction between the extracellular matrix and chondrocytes, which produce a great impact on cell survival. The elution of proteoglycan will be expected to occur primarily from the superficial zone of the cartilage because the water content is the highest and the permeability is superior in the superficial areas [18]. The osmotic responses of chondrocytes may have been exacerbated by the disruption to the extracellular matrix from the scalpel wound where the support structure of the collagen fibers is damaged and the extraction of proteoglycan is accelerated, which is the indirect cause of the chondrocyte death [19].

Chondrocyte metabolism is inordinately inhibited in different osmolarity medium, which may affect cartilage repair. Through the regulation of osmolarity channels, chondrocytes maintain optimal volume and hence mediate any changes in cell metabolism and biosynthesis following exposure to osmotic challenge [20]. As the specific genes in chondrocytes, HIF-1α and type II collagen indicate the metabolic level of chondrocytes. Chondrocytes can be stimulated in hypoxia environment via HIF-1α to express numerous genes associated with chondrocyte anabolism and differentiation, including Sox9, TGF-β, etc., and hypoxia and HIF-1α are associated with type II collagen production [21-23]. The present study indicates that HIF-1α and type II collagen mRNA yields in the primary cultured chondrocytes are the least in 180 mOsm/L medium and are the greatest in 380 mOsm/L, the later closing to the physiological osmolarity of the synovial fluid. Negoro et al. indicated that chondrocytes cultured in closed physiological osmolarity solutions had stronger ability of proteoglycan synthesis in the three-dimensional culture of primary chondrocytes [24].

Saline may not be beneficial to cartilage repair after injury. The cell death in superficial zone, proteoglycan elution, and inhibition of HIF-1α and collagen II expression were significantly less in the balanced salt solutions. By detecting ^35^SO4 uptake rate, Gulihar et al. compared proteoglycan synthesis levels of chondrocyte in different fluids, finding that Ringer’s lactate inhibited cartilage metabolism by 10%, whereas saline caused 35% inhibition [13]. There will be an additional increase in the extracellular sodium ion concentration by the use of saline, potentially leading to altered activity of sodium-dependent membrane transporters, particularly those involved in chondrocyte volume and metabolism [22]. In addition to sodium ion, balanced salt also contains potassium and magnesium ion, etc., which probably supports chondrocyte metabolism better than 0.9% saline that is considered to be not physiological.

Our research still had certain limitations. Firstly, what we chose was the cartilage tissue from knee arthroplasty, but not normal cartilage tissue, which was not easy to obtain. Secondly, the mechanical injury model was steerable, but the real surgical instruments injury was much more intricate. So the influence of osmolarity of irrigation solutions on cartilage repair after injury should be further examined in an *in vivo* model or in a clinical setting. Finally, it needed to further investigate the accurate molecular mechanism, such as the expression and interaction of putative potential osmotic channels (TRPV4 and BKCa) in chondrocytes in different osmolarities [25,26].

## Conclusions

The osmolarity of irrigation solutions plays an important role in the survival and metabolic state of chondrocytes following mechanical injury, and the chondrocyte death is not caused by apoptosis. Increasing osmolarity of irrigation solutions may be chondroprotective with decreasing the chondrocyte death, reducing inhibition of metabolism and proteoglycan elution, ultimately preventing cartilage degeneration and promoting integrative repair.
